# Accurate determination of node and arc multiplicities in de bruijn graphs using conditional random fields

**DOI:** 10.1186/s12859-020-03740-x

**Published:** 2020-09-14

**Authors:** Aranka Steyaert, Pieter Audenaert, Jan Fostier

**Affiliations:** Department of Information Technology, Ghent University-imec, IDLab, Ghent, B-9052 Belgium

**Keywords:** Next-generation sequencing, De Bruijn graphs, Probabilistic graphical models

## Abstract

**Background:**

De Bruijn graphs are key data structures for the analysis of next-generation sequencing data. They efficiently represent the overlap between reads and hence, also the underlying genome sequence. However, sequencing errors and repeated subsequences render the identification of the true underlying sequence difficult. A key step in this process is the inference of the multiplicities of nodes and arcs in the graph. These multiplicities correspond to the number of times each *k*-mer (resp. *k*+1-mer) implied by a node (resp. arc) is present in the genomic sequence. Determining multiplicities thus reveals the repeat structure and presence of sequencing errors. Multiplicities of nodes/arcs in the de Bruijn graph are reflected in their coverage, however, coverage variability and coverage biases render their determination ambiguous. Current methods to determine node/arc multiplicities base their decisions solely on the information in nodes and arcs individually, under-utilising the information present in the sequencing data.

**Results:**

To improve the accuracy with which node and arc multiplicities in a de Bruijn graph are inferred, we developed a conditional random field (CRF) model to efficiently combine the coverage information within each node/arc individually with the information of surrounding nodes and arcs. Multiplicities are thus collectively assigned in a more consistent manner.

**Conclusions:**

We demonstrate that the CRF model yields significant improvements in accuracy and a more robust expectation-maximisation parameter estimation. True *k*-mers can be distinguished from erroneous *k*-mers with a higher F_1_ score than existing methods. A C++11 implementation is available at https://github.com/biointec/detoxunder the GNU AGPL v3.0 license.

## Background

De Bruijn graphs play a key role in many bioinformatics tools as a data structure to efficiently represent the overlap between sequences. Given a set of sequences *S*, the de Bruijn graph’s nodes are defined by the *k*-mers (subsequences of length *k*) present in *S*. Two nodes *u* and *v* are connected by a directed arc when a *k*+1-mer exists in *S* for which the first *k* nucleotides coincide with *u* and the last *k* nucleotides coincide with *v* [1]. Often, linear (i.e. non-branching) chains of nodes are contracted into a single node referred to as a unitig [2].

When *S* corresponds to a set of reads, one obtains the *read-based de Bruijn graph* for which we define the *coverage* of a node (resp. arc) as the number of times its k-mer (resp. *k*+1-mer) occurs in the set of reads. Similarly, when *S* contains a genomic sequence *G*, one obtains the *genome de Bruijn graph* for which we define the *multiplicity* of a node (resp. arc) as the number of times its *k*-mer (resp. *k*+1-mer) occurs in *G*. A read-based de Bruijn graph is a noisy estimate of the (often unknown) genome de Bruijn graph. In real sequencing experiments, read-based de Bruijn graphs contain many spurious nodes/arcs that emerge because of sequencing errors and may be missing nodes/arcs due to coverage gaps. Our goal is to accurately infer the genome de Bruijn graph from its read-based approximation. To this end, we wish to label each node (resp. arc) in the read-based graph with its corresponding multiplicity: true nodes and arcs should be labelled according to their repeat copy number in *G* while spurious nodes/arcs should be assigned multiplicity zero. The multiplicities of nodes and arcs are reflected in their coverage: repeated *k*-mers in the genome often have a higher coverage than non-repeated *k*-mers, while erroneous *k*-mers often have very low coverage.

The accurate inference of the multiplicities of nodes and arcs is non-trivial but nevertheless important: it reveals the sequencing errors (i.e. nodes/arcs that were assigned multiplicity zero) and provides insights into the repeat structure of the genomic sequence. This problem is therefore a common theme in numerous bioinformatics applications, such as *de novo* genome assembly [3], correction of both second and third generation reads [4–7], de Bruijn graph-guided hybrid assembly methods [8–11] and several variant calling and variant-aware assembly methods [12–15].

Using a conditional random field (CRF) model, we demonstrate how local coverage evidence observed at each node or arc individually as well as the evidence of the surrounding neighbourhood can be coupled in a high-dimensional statistical model. Nonetheless, efficient computational methods exist to infer the individual probability distributions for each random variable present in the model. In the last decade, these types of models have been primarily used in the field of computer vision and image analysis, where they facilitated an evolution from heuristic algorithms to a systematic approach by incorporating contextual constraints [16]. We believe that the use of CRFs on de Bruijn graphs can facilitate a similar evolution in the analysis of sequencing data. Using our model, we consistently obtain more accurate multiplicity assignments on both real and simulated data. Moreover, we observe a more robust convergence when learning the model parameters in an expectation-maximisation (EM) setting. Additionally, because we use a statistical framework, we obtain a measure of certainty about the assignments. We believe this methodology can be of use for many tools that rely on de Bruijn graphs. Note that preliminary results of this work have been presented at the HiTSeq workshop of the 2019 ISMB conference [17].

### *k*-mer histogram based approach

**Fig. 1:**
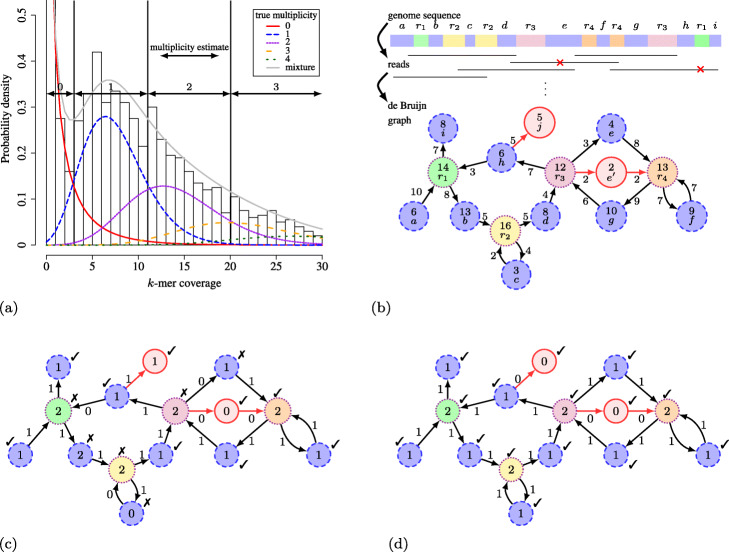
Illustration of the issues when assigning multiplicities based only on a *k*-mer histogram based cutoff. **a** A *k*-mer histogram that consists of a mixture of negative binomial distributions. Each component models the *k*-mer coverage variability for a particular multiplicity. The two-sided arrows delineate the coverage intervals corresponding to a multiplicity estimate. Note how large areas under the curve of a particular multiplicity fall in an interval of a different estimated multiplicity. **b** Example genome sequence with corresponding reads and de Bruijn graph. Nodes and arcs are labelled with their read coverage. Nodes are also labelled with their corresponding fragment in the genome sequence. Sequencing errors cause spurious nodes and arcs in the de Bruijn graph, such as node *e*^′^ and *j*. Nodes are encircled according to their true multiplicity (cf. the patterns in Fig. 1a), all correct arcs have true multiplicity 1. **c** Most likely multiplicity assignment to nodes and arcs based on the *k*-mer histogram in Fig. 1a. This assignment leads to inconsistencies: nodes where there is a conservation of flow of multiplicity are marked with ✓, nodes where this is violated are marked with ✗. **d** Multiplicity assignment such that conservation of flow of multiplicity holds in each node. These assignments are correct for all nodes and arcs and reveal sequencing errors (nodes/arcs with multiplicity zero) and the repeat structure of the genome sequence

Baseline methods for the inference of node and arc multiplicities rely on *k*-mer histograms. A *k*-mer histogram shows, for each *k*-mer coverage, the number of *k*-mers that occur at that coverage in the data. A mixture of distributions is fit to this histogram, where each component corresponds to a distinct multiplicity (see Fig. 1a). Each distribution models, for that multiplicity, the natural variability of coverage that occurs in real sequencing experiments. Using this mixture model, intervals of coverage are selected to point to a certain multiplicity. This idea is adopted by many tools. For example, certain Illumina error correction tools (see [18] for a review) rely on *k*-mer histograms to determine whether or not a *k*-mer is erroneous, i.e. whether or not its coverage falls within the zero-multiplicity interval. Some tools use per-base quality scores to weigh the contribution of each individual *k*-mer occurrence to its coverage [19]. In the context of de Bruijn graphs, the information contained in the *k*-mer histogram is sometimes supplemented with graph-topological features: sequencing errors often emerge in the graph as short dead ends (tips), when the sequencing error occurs towards the end of a read, or as short parallel paths (bubbles), when the error occurs near the centre of a read [20–22].

Nevertheless, as exemplified in Fig. 1, inferring multiplicities from a *k*-mer histogram is error-prone. Figure 1a shows a mixture model of negative binomial (overdispersed Poisson) distributions, each corresponding to a specific multiplicity. Intervals of *k*-mer coverage are established and assigned a particular multiplicity such that the probability for that multiplicity value is maximal in the interval. For example, a *k*-mer with coverage [1…3] is assigned multiplicity zero (sequencing error), coverage interval [4…11] corresponds to multiplicity one (non-repeated sequence), etc. In the example of Fig. 1b, the nodes and arcs of a de Bruijn graph are labelled with a sampled coverage, in order to mimic a sequencing experiment. If we now infer the multiplicities using the intervals of Fig. 1a, we obtain the assignments in Fig. 1c. Not all assignments are correct as some multiplicity estimates are either too high or too low. This is due to the overlap between distributions in the mixture model causing some nodes or arcs with ambiguous coverage (i.e. coverage near the interval boundaries) to be assigned the wrong multiplicity. The selection of hard cutoff intervals will therefore always lead to a certain fraction of incorrect multiplicity assignments, depending on the degree of overlap between distributions, which in turn depends on the sequencing depth and coverage variability of the dataset. To illustrate the practical consequences, a recent survey of Illumina error correction tools [23] demonstrated that many tools suffer from the deletion of low-coverage true *k*-mers, leading to more fragmented assemblies.

### Conservation of flow of multiplicity

Within the context of the de Bruijn graph of Fig. 1c, one can immediately verify the presence of incorrect multiplicity assignments by checking the following criterion at each node: *when all nodes and arcs are correctly labelled with their multiplicity, the multiplicity of each node equals both the sum of the multiplicities of its incoming arcs and the sum of the multiplicities of its outgoing arcs*. This also holds in the presence of sequencing errors as the corresponding spurious nodes or arcs have been assigned multiplicity zero. This property was first noted by Pevzner and Tang [24], who proposed to use maximum-flow algorithms on pre-corrected de Bruijn graphs to determine the multiplicity of repeats. A similar technique has been used on string graphs [25] and on error-free weighted bi-directed graphs [26].

This important property, which we will call *‘conservation of flow of multiplicity’*, is also an important component of our proposed method. Using the conservation of flow property, one might decide that a node or arc with a relatively poor coverage that falls in the zero-multiplicity interval, does have a multiplicity greater than zero because it provides an essential link in the graph. An example of this is the arc between nodes *h* and *r*_1_ in Fig. 1b: *r*_1_ has strong evidence of being a multiplicity 2 repeated node, both by its own coverage and by the sum of the estimated multiplicities of its outgoing arcs. The multiplicities of its incoming arcs should hence also sum up to 2, which can be achieved by assigning multiplicity 1 to the arc from *h* to *r*_1_ despite its low coverage. As a second example, an erroneous *k*-mer with relatively high coverage might still be assigned multiplicity 0 because of flow conservation considerations. In Fig. 1b, once we have established that the arc from node *h* to *r*_1_ should have multiplicity 1, we notice that the arc from *h* to *j* should be assigned multiplicity 0. This further results into erroneous node *j* to also be assigned multiplicity 0. By imposing the conservation of flow property across all nodes and arcs, we obtain the correct multiplicity assignment in Fig. 1d.

## Methods

### Conditional random fields

Conditional random fields (CRFs) are a type of probabilistic graphical models (PGM), i.e. graph models in which the nodes represent random variables and the edges represent direct probabilistic interactions between these variables [27]. Note that this graph is different from the de Bruijn graph, even though both graphs share some resemblance. In case of CRFs, the variables are further divided into a set of observed variables and a set of unobserved variables whose value we want to infer [28]. Here, the observed variables **X**={*X*_1_,…,*X*_*N*_} represent the coverage and the unobserved variables **Y**={*Y*_1_,…,*Y*_*N*_} represent the multiplicities of nodes and arcs in the de Bruijn graph.

Probabilistic interactions are represented by factors *φ*_*i*_(**D**_*i*_), where ${\mathbf {D}_{i} \subseteq \mathbf {X}\cup \mathbf {Y}}, {\mathbf {D}_{i} \nsubseteq \mathbf {X}}$, such that all variables in the scope **D**_*i*_ of *φ*_*i*_ form a clique in the CRF. The joint conditional probability over all variables is then calculated as

$$P(\mathbf{Y}|\mathbf{X}) = \frac{1}{Z(\mathbf{X})}\prod_{i=1}^{M} \varphi_{i}(\mathbf{D}_{i}), $$

with *Z*(**X**) the partition function that normalises the product of factors such that *P*(**Y**|**X**) is a probability distribution [27]. By conditioning on **X**, the modelling of dependencies between these variables can be avoided and only the conditional dependencies between the **Y**s are modelled. We use two types of factors in our model. First, the *‘singleton factors’* *φ* model the relationship between the coverage observed at a node (resp. arc) and its multiplicity. Given the coverage, the factor represents a categorical (also called: multinoulli) distribution over the different possible multiplicities. Second, the *‘conservation of flow factors’* *φ*_flow_ impose the conservation of flow of multiplicity. Such factor has in its scope the multiplicity of a particular node as well as all multiplicities of either incoming or outgoing arcs and assigns a high probability when conservation of flow holds, and a low probability otherwise. They thus model the relationship between the multiplicity of a node and the multiplicities of its adjacent arcs. Collectively, the factors allow evidence (observed coverage) to propagate through the CRF graph. Therefore, the computed multiplicity assignments will be the result of an interplay between locally observed evidence, and evidence observed in the surrounding neighbourhood.

### From de Bruijn graph to CRF

**Fig. 2:**
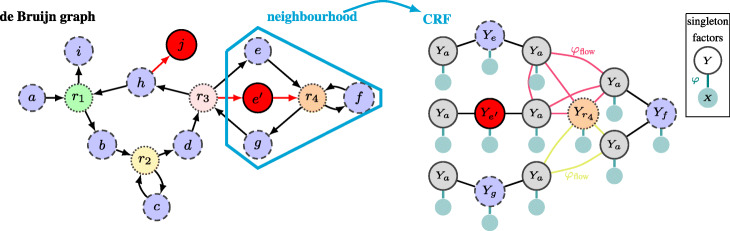
De Bruijn graph of Fig. 1b. In the de Bruijn graph a neighbourhood of size 1 around node *r*_4_ was selected and the CRF corresponding to this neighbourhood is shown. Nodes *Y*_*n*_ in the CRF correspond to nodes *n* in the de Bruijn graph, while the *Y*_*a*_-nodes correspond to the arcs in the de Bruijn graph. Here, each node *Y* is connected to a node *X* by an edge arising from a singleton factor *φ* which contains the information about the coverage of that node or arc in the de Bruijn graph. Note, however, that a node that contains fewer than 2*kk*-mers will not have such corresponding *X* and factor *φ*. All connections between *Y*-nodes arise from conservation of flow factors. The two cliques arising from the flow through *r*_4_ are shown explicitly

Because building a CRF for the entire de Bruijn graph would render computations infeasible for exact inference techniques, CRFs are built for smaller subgraphs: if we want to infer the multiplicity of a particular node *n* in the de Bruijn graph, a neighbourhood of size *s* is selected that consists of all nodes that are reachable from node *n* by a path of length ≤*s* along with all incoming and outgoing arcs of these nodes. For each arc *a* in the neighbourhood, the CRF contains variable nodes *Y*_*a*_ and *X*_*a*_, the multiplicity and observed coverage at that arc, respectively. For each node *m* in this neighbourhood, the CRF contains a variable node *Y*_*m*_, the multiplicity of that node. The average coverage of nodes that contain <2*kk*-mers is highly correlated with the coverage observed at their adjacent arcs. Therefore, we only add a variable node *X*_*m*_ to represent the observed average coverage of node *m* if this node contains more than 2*kk*-mers. Because the time and memory requirements of the subsequent inference algorithm depend on the number of possible values for the variables, we restrict *Val*(*Y*) to [ max(0,*m*_opt_−*α*),*m*_opt_+*α*], where *α* is a tuneable parameter (default value: *α*=2) and *m*_opt_ is the most likely multiplicity for *Y* based on the locally observed coverage *X*. Figure 2 shows a de Bruijn graph with a selected neighbourhood of size 1 and its corresponding CRF.

**Singleton factors** For each *X* and *Y* corresponding to an arc or node in the de Bruijn graph, a singleton factor $\varphi (Y,X): Val(Y,X) \mapsto \mathbb {R}^{+}$ is created that reflects the probability that an arc/node has multiplicity *m* given the observed coverage *C*:

$$\varphi(m,C) = w_{m} P_{m}(X = C), \quad X \sim \text{Negative Binomial} $$

To estimate these probabilities, a mixture model of negative binomial distributions is fitted to the *k*-mer histogram for nodes and the *k*+1-mer histogram for arcs such that each component in the mixture corresponds with a multiplicity *m*. As opposed to Poisson distributions that take only a single parameter *λ* and assume the mean and variance are equal, negative binomial distributions are more flexible as they are able to model data with larger variation. The negative binomial distribution is traditionally parameterised using parameters *r* and *p*, however, in the context of this work, we use parameters $\lambda = \frac {pr}{1-p}$ (mean) and $f = \frac {1}{1-p}$ (overdispersion factor). The variance is then equal to *fλ*. As *p*→0 (in which case *f*→1) and *r*→*∞* such that $\lambda = \frac {pr}{1-p}$ remains constant, the negative binomial distribution converges to a Poisson distribution with mean *λ*.

For *m*=0 the mean of the negative binomial *λ*_0_ is estimated as the mean coverage of the erroneous *k*-mers. We also estimate *λ*, the mean coverage of *k*-mers with multiplicity 1. For each multiplicity *m*≥1 the negative binomial then has mean *mλ*. Additionally, overdispersion factors *f*_0_ for the error-distribution and *f* for the distributions representing *m*≥1 are estimated based on the variance in the data. Finally, each negative binomial is weighted according to the estimated number of *k*-mers with that multiplicity in the dataset, *w*_*m*_.

**Conservation of flow factors** For each *Y*_*n*_ of node *n* in the de Bruijn graph, two conservation of flow factors are created: one for its incoming arcs and one for its outgoing arcs. These factors have in their scope the corresponding CRF-node *Y*_*n*_ and all *Y*_*a*_ corresponding to incoming resp. outgoing arcs of node *n*. E.g. for the incoming arcs:

$$\varphi_{\text{flow}}(m_{n},\{m_{a}\}_{a \in \text{in}(n)}) = \left\lbrace \begin{array}{lr} \!1, & m_{n} = \sum\limits_{a \in \text{in}(n)} m_{a} \\ \!\varepsilon, & m_{n} \neq \sum\limits_{a \in \text{in}(n)} m_{a} \end{array} \right. $$

Factors $\varphi _{\text {flow}}(Y_{n},\{Y_{a}\}_{a \in \text {in}(n)}): Val(Y_{n},\{Y_{a}\}_{a \in \text {in}(n)}) \mapsto \mathbb {R}^{+}$ assign a value of 1 to cases where the sum of multiplicities of the incoming resp. outgoing arcs is equal to the multiplicity of the node and a low value *ε*≪1 (default: 10^−7^) otherwise.

Whereas the singleton factors provide local evidence, the conservation of flow factors direct towards a multiplicity assignment that is consistent with the property of flow of multiplicity for all nodes and arcs in the CRF.

### Inference

The product of all factors is the joint probability of the multiplicities over all nodes and arcs in the selected neighbourhood. To determine the probabilities over the possible multiplicities of a specific node *n* (or arc *a*) we need to calculate $P(Y_{n} | \mathbf {X}) = \sum _{\mathbf {Y}\setminus Y_{n}} P(\mathbf {Y}| \mathbf {X})$. We use the variable elimination algorithm for PGMs with a ‘min-neighbours criterion’ to select the elimination order [27]. This algorithm performs an exact calculation of the probabilities. This solver was implemented in log-space to avoid numerical underflow. Supplementary Section 1, Additional File 1 contains an example.

### Parameter estimation using expectation-maximisation

The parameters of the mixture model need to be estimated from the data. These parameters can be computed using the multiplicities of the nodes/arcs in the de Bruijn graph, which are also unknown. We therefore use the expectation-maximisation (EM) algorithm: during the E-step, the multiplicities of nodes and arcs are inferred using the current model parameter estimates; during the M-step, the parameter values are updated using the newly inferred multiplicities, weighted by their probability. Convergence is obtained when the estimated multiplicity of a pre-specified fraction (default: 0.001) of the nodes and arcs no longer changes between consecutive E-steps.

The parameters of the mixture model are the following: the means of the negative binomial distributions *λ* and *λ*_0_, the overdispersion factors *f* and *f*_0_ that determine the variance, and the weights *w*_*m*_ for all multiplicities. Since nodes correspond to (concatenated) *k*-mers, whereas the arcs correspond to *k*+1-mers, the underlying distributions differ slightly. Therefore, all parameters are estimated separately for the nodes and the arcs of the de Bruijn graph. To avoid complex numerical computations associated with the maximum likelihood estimators, we make use of the method of moment estimators. The exact formulas are provided in Supplementary Section 2, Additional File 1.

The mixture model parameters are estimated using only a small subset of the nodes and arcs in the de Bruijn graph (default: 10.000 nodes and 10.000 arcs). This leads to a significant speedup and still provides accurate estimates for the model parameters.

### Using quality scores

Instead of counting the occurrence of each *k*-mer in the dataset, we use weighted counts based on the quality scores of the nucleotides in the *k*-mer. Such weighted counts are called *q*-mers and were first introduced in the read corrector Quake [19]:

$$q\text{-mer count} = \text{score}(k\text{-mer}) = \prod_{i=1}^{k} qual(x_{i}) $$

Because erroneously called nucleotides often have a low quality score, *k*-mers that overlap with such nucleotides will have a lower *q*-mer count, whereas *k*-mers containing only correct nucleotides will still have a *q*-mer count close to one. As a consequence, the distributions representing the erroneous nodes/arcs and the distributions that represent multiplicity *m*≥1 overlap less.

Note that the negative binomial distribution is a discrete probability distribution and is thus only defined for integer counts. However, by replacing the factorials in the discrete probability mass function (pmf) with Gamma distributions we obtain a pmf that is applicable to continuous data and coincides with the original pmf in the discrete points.

### The full pipeline

BCALM 2 [29] is used to build a compacted de Bruijn graph from raw sequencing data, i.e. a de Bruijn graph in which all linear paths have been concatenated into single nodes called unitigs. BCALM 2 is capable of constructing de Bruijn graphs even for large genomes with relatively low memory requirements. For all results presented in this paper we use *k*-mer size *k*=21. Unless mentioned otherwise, we use BCALM 2 with the -abundance-min=2 option to remove all *k*-mers that occur only once in the reads. BCALM 2 outputs a de Bruijn graph which serves as input for Detox, the tool in which we implemented the proposed CRF model. Detox uses three stages. In stage 1, the *q*-mer (or optionally: *k*-mer) coverages of all nodes and arcs in the graph are computed. In the stage 2, the mixture model parameters are estimated using the expectation-maximisation algorithm. Finally, in stage 3, the multiplicities of all nodes and arcs in the de Bruijn graph are inferred using CRFs. These multiplicities are written to disk. Detox is implemented in C++11 and uses threads for parallelisation. We refer to Supplementary Section 7, Additional File 1 for a discussion of the choice and default value of the most important input parameters.

## Results

### Worked example

**Fig. 3:**
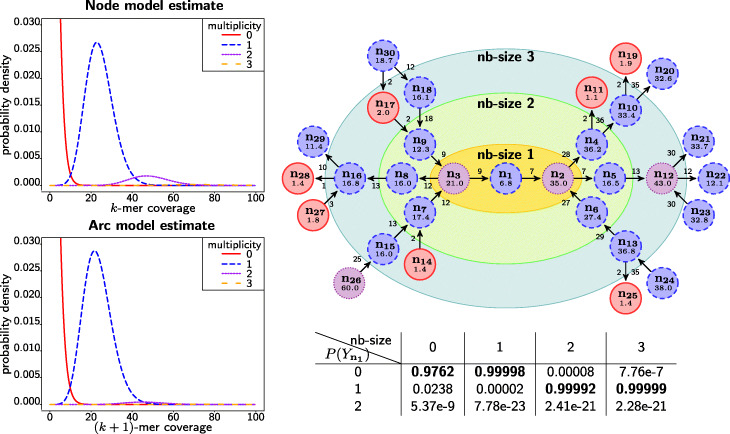
Subgraph of the de Bruijn graph built from the real P. aeruginosa dataset (30×). Nodes and arcs are labelled with their (average) coverage. The fitted node and arc models are shown on the left. The nodes of the graph are coloured according to their true multiplicity. The neighbourhoods of size 1, 2 and 3 surrounding node *n*_1_ are shown as coloured ellipses. The inset table shows the categorical distribution $P(Y_{\mathbf {n_{1}}})$ of the multiplicities {0,1,2} for node *n*_1_ for different neighbourhood sizes. For small neighbourhoods (sizes 0 and 1), node *n*_1_ (incorrectly) appears to represent a sequencing error due to its relatively low coverage. At neighbourhood sizes 2 and 3, the CRF model has enough information to (correctly) infer multiplicity 1 for node *n*_1_ with high probability

To gain some intuition, we present a specific case in which the CRF framework outperforms a *k*-mer cutoff methodology. Figure 3 shows a subgraph of the de Bruijn graph, built from the *P. aeruginosa* dataset (real data, subsampled to 30×). We infer the multiplicity of node *n*_1_ (with a true multiplicity of 1) using our proposed CRF methodology for different neighbourhood sizes *s*. At neighbourhood size *s*=0, the inferred multiplicity is solely based on the (average) *k*-mer coverage observed at node *n*_1_ and hence, the CRF method degenerates to the *k*-mer cutoff methodology. With larger neighbourhood sizes (*s*≥1), the CRF model also incorporates the coverage observed at nodes (and their adjacent arcs) in the neighbourhood of node *n*_1_. After applying the variable elimination algorithm, we obtain a categorical (multinoulli) distribution over the different multiplicities for node *n*_1_ (see inset table of Fig. 3). The estimated multiplicity is then simply the one with the highest probability. These probabilities thus provide a measure of confidence.

The issue in the subgraph of Fig. 3 is that part of the path of the genome through *n*_18_,*n*_9_,*n*_3_,*n*_1_,*n*_2_,*n*_5_ has low coverage. If we only consider individual coverages, node *n*_1_ incorrectly appears to be a sequencing error whereas its true multiplicity is 1. Similarly, nodes *n*_2_ and *n*_3_ appear to have multiplicity 1 whereas their true multiplicity is 2. Because all three nodes suffer from low coverage, a CRF with a neighbourhood of size *s*=1 does not yet improve the multiplicity estimation. On the contrary, it increases the probability of a zero multiplicity for node *n*_1_ because the evidence of a multiplicity of 1 for nodes *n*_2_ and *n*_3_ is consistent with a zero multiplicity for node *n*_1_. However, when using a neighbourhood of size *s*≥2, the CRF also incorporates information about parts of the path with higher coverage. Particularly, the coverage observed at arc *a*_18→9_ suggests a multiplicity 1 for node *n*_9_ (which itself has ambiguous coverage) and hence, in combination with evidence for multiplicity 1 for node *n*_7_, a multiplicity 2 for node *n*_3_. Similarly, it becomes clear that also node *n*_2_ has multiplicity 2. Ultimately, a consistent multiplicity assignment can only be obtained when node *n*_1_ is (correctly) assigned a multiplicity of 1. As we incorporate even more information at neighbourhood size *s*=3, this belief is further strengthened and the CRF model infers the correct multiplicity with a high degree of confidence.

### Multiplicity assignment performance

To evaluate the overall performance of the proposed CRF methodology, we consider five real Illumina datasets (two bacterial and three eukaryotic – see Table 1). To assess the impact of coverage depth, each dataset was downsampled to different coverage depths of 10×,25× and 50×. Using BCALM 2, compacted de Bruijn graphs (*k*=21) were built for the 15 resulting datasets. BCALM 2 was configured to discard *k*-mers that occur only once in the data. As such, we obtained de Bruijn graphs with a number of nodes ranging between 20 220 (*S. enterica*, 10×) and 254.7 million (*H. sapiens*, 50×). For all nodes and arcs, the quality score weighted coverage was computed.

**Table 1:** Illumina datasets used in this paper

Organism	Reference ID	Ref. size	Accession no.	Model	Read length	Reference
*S. enterica*	NC_011083.1	5.1 Mb	SRR1206093	MiSeq	2×250 bp	[30]
*P. aeruginosa*	NC_002516.2	6.3 Mb	ERR330008	MiSeq	2×150 bp	[31]
*C. elegans*	WBcel235	100.3 Mb	SRR5865874	HiSeq 2000	2×100 bp	[32]
*A. thaliana*	TAIR10.1	119.4 Mb	ERR1913320	NextSeq 500	2×150 bp	-
*H. sapiens*	GRCh38	3099.8 Mb	ERR194147	HiSeq 2000	2×100 bp	[33]

We inferred the node multiplicities using the CRF methodology for different neighbourhood sizes *s* (0, 1, 3 and 5). For all organisms, except *H. sapiens*, we inferred the multiplicity of all nodes in the graph. To avoid excessive runtimes, the multiplicity for *H. sapiens* was inferred for one million randomly sampled nodes. Table 2 shows the node (resp. *k*-mer) accuracy as the percentage of nodes (resp. *k*-mers) that were assigned the correct multiplicity. For all organisms and for all coverage depths, the CRF methodology (neighbourhood size *s*>0) outperforms the baseline methodology (neighbourhood size *s*=0) by a significant margin. With increasing neighbourhood size *s*, the accuracy increases with which CRFs assign multiplicities. Most of this gain is already realised for small values of *s* (e.g. *s*=1), indicating that the coverage information of nodes and arcs within close proximity of a node (resp. arc) is most informative to correctly assign the multiplicity of that node (resp. arc).

**Table 2:** Estimation of the node multiplicity in de Bruijn graphs (*k*=21) built from real Illumina data for 5 organisms (2 bacteria, 3 eukaryotes)

		10×			25×			50×	
	*s*	node acc.	*k*-mer acc.		node acc.	*k*-mer acc.		node acc.	*k*-mer acc.
*P. aeruginosa*	0	84.31	96.47		95.02	98.72		97.47	99.21
	1	92.85	98.80		98.27	99.49		98.89	99.46
	3	93.91	98.96		98.60	99.50		99.13	99.52
	5	94.11	98.94		98.74	99.51		99.17	99.51
*S. enterica*	0	84.50	96.46		93.65	98.25		95.96	98.53
	1	88.81	97.18		94.98	98.39		96.44	98.60
	3	89.41	97.18		95.27	98.45		96.53	98.63
	5	89.55	97.22		95.32	98.46		96.57	98.64
*C. elegans*	0	68.65	93.93		80.69	96.42		87.35	97.10
	1	78.90	96.48		86.47	97.77		90.74	98.05
	3	81.02	97.21		87.16	98.01		91.27	98.24
	5	81.29	97.18		87.32	98.05		91.25	98.25
*A. thaliana*	0	67.84	89.10		82.20	96.16		89.91	97.05
	1	73.67	94.64		85.45	96.83		91.27	97.54
	3	73.92	95.26		85.83	97.09		91.46	97.71
	5	73.93	95.43		85.68	97.17		91.56	97.70
*H. sapiens*	0	75.26	92.27		83.29	94.67		88.09	95.51
	1	80.68	93.92		85.66	95.23		89.23	95.83
	3	81.33	94.56		86.12	95.50		89.52	95.95
	5	81.57	94.71		86.26	95.58		89.59	95.97

The datasets were downsampled to coverage depths of 10×,25× and 50×. For *H. sapiens*, the multiplicity was inferred for one million randomly sampled nodes; for all other datasets the multiplicity was inferred for all nodes. The node (resp. *k*-mer) accuracy refers to the percentage of nodes (resp. *k*-mers) in the de Bruijn graph that were assigned the correct multiplicity. The accuracy improves when using CRFs with increasing neighbourhood size *s*

With higher coverage depth, there is less overlap between the coverage distributions associated with the different multiplicities and hence, node multiplicities can be assigned more easily. The CRF method therefore proves especially useful at low or moderate coverage depths. For example, for *C. elegans* at 10×, the node accuracy improves from 68.65*%* (*s*=0) to 81.29*%* (*s*=5), an improvement of 12.64 percentage points. Nevertheless, even at 50× coverage depth, the use of CRFs yield improvements in node accuracy of 1.5 to 4 percentage points.

In all cases, the *k*-mer accuracy is higher than the node accuracy (Table 2). Inferring the multiplicity is easier for nodes that contain more *k*-mers, or in other words, nodes that represent longer unitig sequences. In those cases, the coverage of a node is averaged over a larger number of *k*-mers, and hence, due to the central limit theorem, it has a higher probability of being closer to the mean coverage for the corresponding multiplicity.

Correctly inferring the multiplicity becomes increasingly difficult for higher multiplicity values (Supplementary Tables 1 and 5, Additional File 1). In Supplementary Table 1, Additional File 1, we provide confusion matrices for the different organisms, coverage depths (10× and 50×) and neighbourhood sizes (*s*=0 and *s*=5). Each column of a confusion matrix corresponds to all nodes with a specific true multiplicity (0 to 5) and shows how the estimated multiplicities are distributed. For all organisms, the majority of nodes in the de Bruijn graph represents either a sequencing error or a non-repeated true sequence. Especially for those nodes, estimating the multiplicity appears relatively easy. However, the correct multiplicity inference for nodes that represent repeated sequences is difficult, even at a high coverage depth. For those nodes, the use of CRFs significantly boosts accuracy. For example, for *C. elegans* (50 ×), without the CRF method (*s*=0) only 54.5% (267 985 out of 429 137) of the nodes with true multiplicity 2 are correctly classified; this figure increases to 83.7% (411 764 out of 429 137) with CRFs (*s*=5). Hence, even if the coverage of a repeated node is ambiguous (or even misleadingly high or low), the CRF method can often still correctly infer the correct multiplicity from its surrounding context.

We also assessed the proposed CRF model using simulated Illumina data that was generated using the ART tool. We used the same organisms, coverage depths and parameter settings as for the real data. Supplementary Table 4, Additional File 1 shows the node and *k*-mer accuracy. Again, the use of CRFs leads to improved multiplicity estimations in all cases and this effect increases with increasing values for the neighbourhood size *s*. In general, the accuracies obtained on simulated data are higher than those obtained for real data. There are several reasons for this. Real data may suffer from coverage biases that are not fully captured by read simulation tools. More importantly, establishing the ground-truth node and arc multiplicities is more difficult for real data. Due to the presence of genetic variation (single nucleotide polymorphism, copy number or structural variation), the sequenced individuals may not be a 100% match with their reference genome. Moreover, the samples may include reads that originate from extra-chromosomal DNA (e.g. a plasmid in *A. thaliana*) for which the ground-truth multiplicity labelling cannot accurately be done using the reference genome. For example, from the confusion matrix for *S. enterica* (Supplementary Table 1, Additional File 1), one observes 2779 nodes with an estimated multiplicity >5 but for which the true multiplicity is 0. Clearly, these nodes represent DNA that is present in the sample, but not in the reference genome. One should take these considerations into account when interpreting the accuracy values in Table 2.

As a final note, the use of the CRF model reduces the number of iterations needed for the EM algorithm to converge (see Supplementary Tables 2-3, Additional File 1). During the E-step, node and arc multiplicities are inferred using the current estimation of the model parameters. Subsequently, during the M-step, the model parameters are updated. Clearly, more accurate multiplicity inference during the M-step leads to a more rapid convergence. In certain cases, we found that the EM-algorithm did not converge when *not* using the CRF methodology (*s*=0), e.g., for *H. sapiens* (10 ×). In that case, the distributions that correspond to the error nodes and multiplicity 1 nodes are difficult to distinguish. In contrast, when using CRFs (neighbourhood sizes *s*>1), the EM-algorithm always converged to the correct model, even for low coverage depths.

### Comparison to existing methods

We are unaware of existing tools to determine multiplicities of nodes and arcs in de Bruijn graphs. To compare with existing methods we consider a subproblem of multiplicity determination, i.e. the classification of *k*-mers into trusted (multiplicity *m*≥1) and untrusted (multiplicity *m*=0) *k*-mers. This task is an essential subtask of many error correction tools.

Quake [19] discerns trusted from untrusted *k*-mers by fitting a mixture of distributions to the *q*-mer histogram. Next, a *q*-mer cutoff value is determined below which a *k*-mer has an *α* times higher chance to belong the error distribution than to the true distribution. We ran Quake v.0.3.5 with default parameters to produce a coverage cutoff value and then used Jellyfish v.1.1.12 to partition the *k*-mers into trusted and untrusted subsets. We also compare to BayesHammer [34], a method that uses sequence similarity and Bayesian subclustering techniques on a Hamming graph of *k*-mers. Trusted *k*-mers are determined as the centre of these clusters. We used BayesHammer as a submodule of SPAdes v.3.13.1 with default parameters.

For each method, we compute the number of true positives (TP, erroneous *k*-mers correctly classified), true negatives (TN, true *k*-mers correctly classified), false positives (FP, true *k*-mers incorrectly labelled as untrusted) and false negatives (FN, erroneous *k*-mers incorrectly labelled as trusted). We then compute the sensitivity, specificity and F_1_ score as follows:

$$\begin{array}{*{20}l} & \text{sensitivity} = \frac{\text{TP}}{\text{TP}+\text{FN}} \\ & \text{specificity} = \frac{\text{TN}}{\text{TN}+\text{FP}} \\ & \mathrm{F}_{1} \text{ score} = \frac{2\text{TP}}{2\text{TP}+\text{FP}+\text{FN}} \end{array} $$

Table 3 shows the sensitivity, specificity and F_1_ score obtained by Quake, BayesHammer and our proposed CRF method (Detox). At 10× coverage, BCALM 2 was configured to retain *allk*-mers as we noted that filtering the *k*-mers that occur only once caused a significant number of false positives. Also note that for *H. sapiens*, only the reads that map to chromosome 21 were used because BayesHammer required more memory than the available 256 GB RAM to handle human genome scale datasets.

**Table 3:** Sensitivity, specificity and F_1_ score for the classification of *k*-mers into trusted and untrusted subsets

	10× cov				25× cov				50× cov		
	sens	spec	F_1_		sens	spec	F_1_		sens	spec	F_1_
	*S. enterica*										
Quake	0	**100**	0		93.77	**100**	96.78		99.31	99.97	99.65
BayesHammer	98.22	99.42	98.81		99.22	99.98	99.60		99.42	**99.98**	99.71
Detox	**98.46**	99.21	**98.82**		**99.51**	99.95	**99.74**		**99.75**	99.96	**99.87**
	*P. aeruginosa*										
Quake	4.64	**100**	8.88		80.58	**100**	89.24		98.08	**100**	99.03
BayesHammer	99.07	99.64	99.11		99.25	99.99	99.62		99.23	**100**	99.61
Detox	**99.23**	99.69	**99.25**		**99.80**	99.97	**99.88**		**99.90**	99.97	**99.94**
	*C. elegans*										
Quake	n/a	n/a	n/a		n/a	n/a	n/a		91.68	99.94	95.65
BayesHammer	96.64	**98.71**	97.40		96.49	**99.88**	98.18		96.34	**99.95**	98.13
Detox	**99.20**	98.96	**98.87**		**99.81**	99.69	**99.82**		**99.91**	99.68	**99.91**
	*A. thaliana*										
Quake	0	**100**	0		94.20	**99.99**	97.01		99.00	**99.96**	99.49
BayesHammer	96.34	98.52	96.93		95.88	99.84	97.84		95.11	**99.96**	97.49
Detox	**98.36**	97.68	**97.68**		**99.42**	99.40	**99.49**		**99.70**	99.54	**99.76**
	*H. sapiens chr. 21*										
Quake	n/a	n/a	n/a		25.06	99.46	39.88		48.96	**99.86**	65.7
BayesHammer	84.62	**99.05**	90.53		88.9	**99.79**	94.01		90.8	99.84	95.12
Detox	**90.09**	98.39	**92.88**		**95.84**	98.47	**97.04**		**97.5**	98.41	**98.2**

Quake and BayesHammer were run with default settings. Our CRF method (Detox) was run at neighbourhood size *s*=3. For C. elegans at 10× and 25× coverage and H. sapiens, chr. 21, Quake was unable to identify a cutoff and hence, no results are shown (n/a). The highest values are shown in boldface

**Fig. 4:**
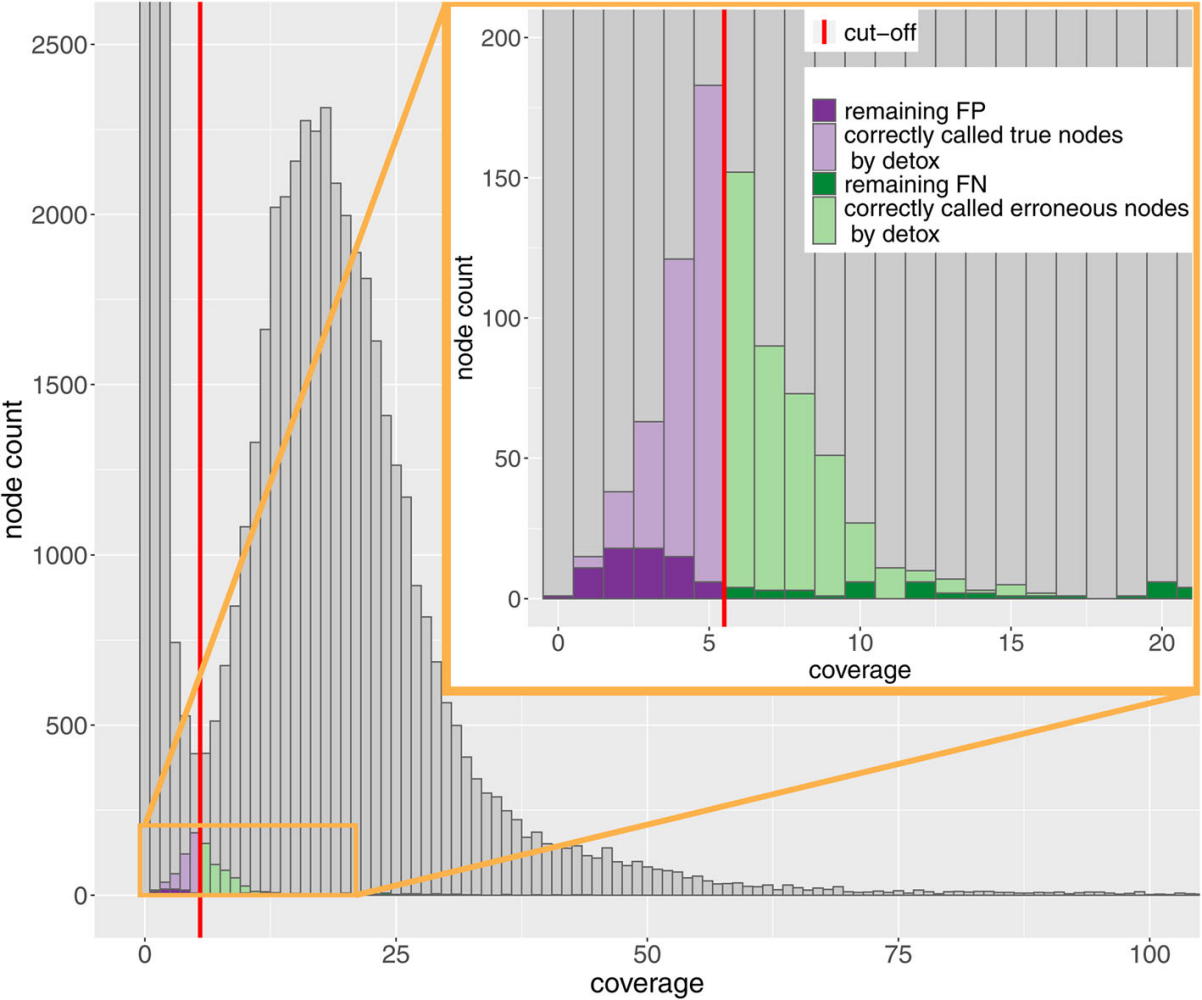
Part of the *q*-mer histogram of the real *P. aeruginosa* (25×) dataset. The red line shows the optimal coverage cutoff value. The purple shaded area represents nodes with a coverage below this cutoff value that have true multiplicity 1 whereas the green shaded area represents nodes with a coverage above the cutoff that have true multiplicity 0. These nodes represent false positives and false negatives respectively when using a cutoff-based method. In contrast, the CRF model is able to correctly classify the majority of nodes (lighter shaded purple and green areas)

For all organisms and coverage depths, Detox attains the highest F_1_ score. Both Quake and BayesHammer appear to be conservative methods: they have a high specificity at the cost of a low sensitivity. For Quake, a higher *q*-mer cutoff value would result in a higher sensitivity, at the cost of a lower specificity. However, we emphasise that the higher F_1_ score for Detox is not solely due to the fact that Quake may select a (too) low *q*-mer cutoff value. This is illustrated in Fig. 4, where we zoom in on the *q*-mer histogram of the *P. aeruginosa* 25× coverage dataset. The red line shows the *q*-mer cutoff point that yields the highest F_1_ score. Methods such as Quake classify *allk*-mers with a coverage below this cutoff value as untrusted and *all* other *k*-mers as trusted. In contrast, our CRF model is able to infer that certain *k*-mers with a coverage below the cutoff value have a true multiplicity =1. Conversely, the CRF model can deduce that *k*-mers with a coverage above the cutoff value correspond to sequencing errors. In other words, a node that is assigned multiplicity zero might have a higher coverage than a node that is assigned multiplicity one. This unique property explains the higher classification accuracy obtained by Detox. Indeed, Fig. 4 illustrates that most *k*-mers with ambiguous coverage are correctly classified.

### Runtime performance

The runtime of the CRF methodology to infer the multiplicity of a node (or arc) depends on the total number of nodes and arcs in its neighbourhood as well as the degree of connectivity of this subgraph. Linear subgraphs give rise to CRFs that can be solved in a time proportional to the number of nodes and arcs in the subgraph, whereas densely connected subgraphs yield CRFs that may require a solution time that is exponential in the total number of nodes and arcs. We emphasise, however, that for a *fixed* value of the neighbourhood size *s*, the total number of nodes and arcs in a subgraph is bounded, and hence, the runtime to infer the multiplicity for a particular node or arc is O(1). Therefore, the runtime to infer the multiplicity of all nodes (resp. arcs) in de Bruijn graph scales linearly with the number of nodes (resp. arcs).

Table 4 lists the average number of nodes and arcs in a subgraph for different genomes (50× coverage depth) and different neighbourhood sizes *s* as well as the total runtime to infer the multiplicity for 10 000 randomly selected nodes using a single core of a 24-core AMD EPYC 7451 CPU with a base clock frequency of 2.3 GHz. With increasing neighbourhood size *s*, the number of nodes and arcs in the subgraph increases rapidly. Genomes with a complex repeat structure such as *H. sapiens* give rise to larger and more densely connected subgraphs for which the corresponding CRF solution requires a longer runtime.

**Table 4:** Performance assessment for multiplicity inference using CRFs

		avg. no.	avg. no.	runtime
	*s*	of nodes	of arcs	(10k nodes)
*P. aeruginosa*	0	1.0	0.0	0.024 s
	1	3.1	6.0	0.566 s
	3	11.9	20.8	3.5 s
	5	28.7	49.4	18.0 s
*C. elegans*	0	1.0	0.0	0.028 s
	1	3.8	10.8	1.292 s
	3	23.7	66.1	46.3 s
	5	77.1	210.0	257 s
*H. sapiens*	0	1.0	0.0	0.047 s
	1	4.4	14.4	1.974 s
	3	32.7	103.9	76 s
	5	115.0	345.7	605 s

For different organisms (at 50× coverage depth) and neighbourhood sizes *s*, the average number of nodes and arcs in a neighbourhood are listed as well as the runtime to infer the multiplicity of 10000 nodes using a single CPU thread

Recall that most of the accuracy gains of the CRF methodology are already obtained for *s*=1 (see Table 2). In that case, the CRF includes coverage information of, depending on the genome, 3–5 nodes and 6–15 arcs. Using all 24 cores of the AMD EPYC 7451 CPU, the multiplicity of all 254.7 million nodes in the *H. sapiens* de Bruijn graph (50× coverage depth) can be inferred in roughly 1 h and 20 min, illustrating that the CRF methodology is applicable to even the most challenging genomes. For such large-scale de Bruijn graphs, the use of higher values for *s* should likely be restricted to a subset of nodes, e.g. the nodes for which the multiplicity is ambiguous, to avoid excessive runtimes. Bacterial genomes do not suffer from this restriction. Even for *s*=5, the multiplicity of all 169 028 nodes in the de Bruijn graph of *P. aeruginosa* can be inferred in only 27.2 s, using 24 CPU cores.

## Discussion

Many genome analysis tools such as *de novo* genome assemblers, read correction tools and variant callers rely on de Bruijn graphs to represent the underlying genomic sequence. To obtain an informative de Bruijn graph representation, it is important to accurately determine the multiplicities of its nodes and arcs. Nodes/arcs that are assigned multiplicity zero reveal sequencing errors, while higher-order multiplicities provide insight into the repeat structure of the graph.

We used conditional random fields (CRFs) to incorporate contextual information on neighbouring nodes/arcs in a de Bruijn graph and demonstrated that the CRF model significantly improves the accuracy with which multiplicities are assigned for a wide range of organisms and different coverage depths. For the specific subtask of classifying *k*-mers into trusted and untrusted subsets, the CRF model outperformed two existing methods: one based on *q*-mer histograms and one based on clustering of *k*-mers by sequence similarity. Moreover, the use of CRFs in an EM setting provides for a robust estimation of the parameters of the distributions that underlie the *k*-mer or *q*-mer histogram.

Several improvements to the CRF model can be considered as future work. First, the CRF model currently does not take the ploidy of the underlying genome into account. In the case of a diploid organism such as *H. sapiens*, *k*-mers that represent heterozygous variants should be assigned a multiplicity of one half. In the current implementation, the flow of conservation rule forces the multiplicity of one allele to one and the other allele to zero. Second, the computational performance of the method could likely be improved. We demonstrated that the use of neighbourhood size *s*=1 is feasible for human genome scale datasets, however, runtime increases rapidly for larger values of *s*. Several options exist. One may consider a dynamic selection of *s*, where larger values for *s* are used only for a subset of nodes/arcs whose accurate multiplicity determination is difficult (e.g. because of ambiguous coverage) or crucial (e.g. during repeat resolution) and resort to smaller values of *s* (even *s*=0) for the cases where the multiplicity can be unambiguously derived from the locally observed coverage. Alternatively, the use of approximate inference techniques could be researched. As opposed to the variable elimination algorithm, approximate inference techniques may not yield exact numerical results, but can be more computationally efficient.

## Conclusions

In this paper, we provided a method to improve one particular subtask of sequence analysis using de Bruijn graphs. It remains to be demonstrated that more accurate multiplicity determination can also lead to improved practical bioinformatics tools. We plan to develop a de Bruijn graph cleaning tool that makes use of the multiplicities inferred by the CRF model. In combination with an accurate sequence-to-graph alignment tool, this should yield highly accurate short read or hybrid long read error correction tools. Finally, it would be interesting to investigate two what extent accurate multiplicity estimates could improve the repeat resolution step during de novo genome assembly.

## Supplementary information

**Additional file 1** Supplementary material: accurate determination of multiplicities of nodes and arcs in a de bruijn graph. This document contains a worked example of the Variable Elimination algorithm in which we also highlight how the CRF improves multiplicity assignments by using contextual information. It also contains our parameter estimation formulas as well as some additional Figures and Tables referred to in the main text. Finally we present an exploration of the influence of two important parameters in our methodology: the ‘conservation of flow strength’ and the size of the subset used for EM training in stage 2.

## Data Availability

The data that support the findings of this study are publicly available. Table 1 in the manuscript lists the data set identifiers and references for the data that supports the results in the manuscript. A C++11 implementation is available at https://github.com/biointec/detoxunder the GNU AGPL v3.0 license.

## Abbreviations

CRF: Conditional random field
EM: Expectation-maximisation
FN: False negative
FP: False positive
PGM: Probabilistic graphical model
pmf: Probability mass function
TN: True negative
TP: True positive
