# Supplementary material for: Accurate determination of node and arc multiplicities in de bruijn graphs using conditional random fields
Source: BMC Bioinformatics. 2020 Sep 14;21:402. doi: 10.1186/s12859-020-03740-x (PMC7491180; doi:10.1186/s12859-020-03740-x)
Supplement: Supplementary file 1 — Additional file 1 Supplementary material: accurate determination of multiplicities of nodes and arcs in a de bruijn graph. This document contains a worked example of the Variable Elimination algorithm in which we also highlight how the CRF improves multiplicity assignments by using contextual information. It also contains our parameter estimation formulas as well as some additional Figures and Tables referred to in the main text. Finally we present an exploration of the influence of two important parameters in our methodology: the ‘conservation of flow strength’ and the size of the subset used for EM training in stage 2. [file 12859_2020_3740_MOESM1_ESM.pdf]

Supplementary material: accurate determination of multiplicities of nodes and arcs in a de Bruijn graph

Aranka Steyaert, Pieter Audenaert and Jan Fostier

June 11, 2020

1 Variable elimination: worked-example

We provide step-by-step calculations used in the variable elimination algorithm for the example in Figure 3 in the main text. To represent factors, we adopt the matrix-like notation from [1].

Each node and arc in the graph of Main Text: Figure 3 is labelled with its (average) coverage. The smallest possible neighbourhood in our pipeline, i.e. one of size 1, contains nodes $\mathbf{n_1}, \mathbf{n_2}$ and $\mathbf{n_3}$, together with all their incoming and outgoing arcs. However, for the sake of simplicity of this example we will use the CRF framework to obtain the belief in the multiplicity of node $\mathbf{n_1}$ based on the observed coverage in node $\mathbf{n_1}$ and its incoming and outgoing arcs. Thus, we create singleton factors for arcs $\mathbf{a_{3\to1}}$ and $\mathbf{a_{1\to2}}$ based on their respective arc coverages and the arc model. As node $\mathbf{n_1}$ contains 284 concatenated k-mers, a singleton-factor is created for $\mathbf{n_1}$ based on its *average* coverage and the node model:

$$\varphi_{\mathbf{a_{3\to1}}}(Y_{\mathbf{a_{3\to1}}}) = \begin{pmatrix} Y_{\mathbf{a_{3\to1}}} & \varphi_{\mathbf{a_{3\to1}}} \\ 0 & 0.6902 \\ 1 & 0.3098 \\ 2 & 2.19\text{e-}7 \end{pmatrix} \quad \varphi_{\mathbf{n_1}}(Y_{\mathbf{n_1}}) = \begin{pmatrix} Y_{\mathbf{n_1}} & \varphi_{\mathbf{n_1}} \\ 0 & 0.9762 \\ 1 & 0.0238 \\ 2 & 5.37\text{e-}9 \end{pmatrix} \quad \varphi_{\mathbf{a_{1\to2}}}(Y_{\mathbf{a_{1\to2}}}) = \begin{pmatrix} Y_{\mathbf{a_{1\to2}}} & \varphi_{\mathbf{a_{1\to2}}} \\ 0 & 0.9519 \\ 1 & 0.0481 \\ 2 & 1.11\text{e-}8 \end{pmatrix}.$$

Each factor represents a categorical distribution (also called multinoulli distribution) over the different multiplicities: for each multiplicity (first column) its probability is provided (second column). In this case, all three factors put the highest belief in a multiplicity = 0.

The singleton-factors are connected by flow factors that express the conservation of flow of multiplicity. In this particular example, the flow factors corresponding with node $\mathbf{n_1}$ express that the arc multiplicity should equal the node multiplicity:

$$\varphi_{\text{flow}}(Y_{\mathbf{a_{3\to1}}}, Y_{\mathbf{n_1}}) = \begin{pmatrix} Y_{\mathbf{a_{3\to1}}} & Y_{\mathbf{n_1}} & \varphi_{\text{flow}} \\ 0 & 0 & 1 \\ 0 & 1 & \varepsilon \\ 0 & 2 & \varepsilon \\ 1 & 0 & \varepsilon \\ 1 & 1 & 1 \\ 1 & 2 & \varepsilon \\ 2 & 0 & \varepsilon \\ 2 & 1 & \varepsilon \\ 2 & 2 & 1 \end{pmatrix} \qquad \varphi_{\text{flow}}(Y_{\mathbf{a_{1\to2}}}, Y_{\mathbf{n_1}}) = \begin{pmatrix} Y_{\mathbf{a_{1\to2}}} & Y_{\mathbf{n_1}} & \varphi_{\text{flow}} \\ 0 & 0 & 1 \\ 0 & 1 & \varepsilon \\ 0 & 2 & \varepsilon \\ 1 & 0 & \varepsilon \\ 1 & 1 & 1 \\ 1 & 2 & \varepsilon \\ 2 & 0 & \varepsilon \\ 2 & 1 & \varepsilon \\ 2 & 2 & 1 \end{pmatrix}$$

where ε is a value $\ll 1$. This way a high belief is expressed in arc and node multiplicity combinations that agree with a conservation of flow of multiplicity and a low belief otherwise. The strength with which we impose conservation of flow of multiplicity is determined by ε. We then multiply all factors and marginalise over (i.e. sum out) the arc variables and normalise to obtain the marginal distribution of $Y_{\mathbf{n_1}}$:

$$P(Y_{\mathbf{n_1}}) = \frac{1}{Z} \sum_{Y_{a_{1\to2}}} \sum_{Y_{a_{3\to1}}} \varphi_{\mathbf{a_{3\to1}}}(Y_{\mathbf{a_{3\to1}}}) \cdot \varphi_{\text{flow}}(Y_{\mathbf{a_{3\to1}}}, Y_{\mathbf{n_1}}) \cdot \varphi_{\mathbf{n_1}}(Y_{\mathbf{n_1}}) \cdot \varphi_{\text{flow}}(Y_{\mathbf{a_{1\to2}}}, Y_{\mathbf{n_1}}) \cdot \varphi_{\mathbf{a_{1\to2}}}(Y_{\mathbf{a_{1\to2}}})$$

where Z is a normalisation constant. If we were to multiply all factors before summing out the relevant variables, the obtained intermediate factor would become very large. The variable elimination algorithm instead eliminates these variables one by one. We first eliminate variable $Y_{\mathbf{a_{1\to 2}}}$:

$$P(Y_{\mathbf{n_1}}) = \frac{1}{Z} \sum_{Y_{a_{3\to 1}}} \varphi_{\mathbf{a_{3\to 1}}}(Y_{\mathbf{a_{3\to 1}}}) \cdot \varphi_{\text{flow}}(Y_{\mathbf{a_{3\to 1}}}, Y_{\mathbf{n_1}}) \cdot \varphi_{\mathbf{n_1}}(Y_{\mathbf{n_1}}) \cdot \underbrace{\sum_{Y_{a_{1\to 2}}} \varphi_{\mathbf{a_{1\to 2}}}(Y_{\mathbf{a_{1\to 2}}}) \cdot \varphi_{\text{flow}}(Y_{\mathbf{a_{1\to 2}}}, Y_{\mathbf{n_1}})}_{\tau_1(Y_{\mathbf{n_1}})}$$

To calculate τ_1 we multiply factors φ_{flow} and $\varphi_{\mathbf{a_{1\to 2}}}$ an then marginalise over $Y_{\mathbf{a_{1\to 2}}}$ as follows:

$$\sum_{Y_{a_{1\to 2}}} \begin{pmatrix} Y_{\mathbf{a_{1\to 2}}} & Y_{\mathbf{n_1}} & \varphi_{\text{flow}} \\ 0 & 0 & 1 \\ 0 & 1 & \varepsilon \\ 0 & 2 & \varepsilon \\ 1 & 0 & \varepsilon \\ 1 & 1 & 1 \\ 1 & 2 & \varepsilon \\ 2 & 0 & \varepsilon \\ 2 & 1 & \varepsilon \\ 2 & 2 & 1 \end{pmatrix} \cdot \begin{pmatrix} Y_{\mathbf{a_{1\to 2}}} & \varphi_{\mathbf{a_{1\to 2}}} \\ 0 & 0.9519 \\ 1 & 0.0481 \\ 2 & 1.11\text{e-}8 \end{pmatrix} = \sum_{Y_{a_{1\to 2}}} \begin{pmatrix} Y_{\mathbf{a_{1\to 2}}} & Y_{\mathbf{n_1}} & \varphi_{\text{flow}} \cdot \varphi_{\mathbf{a_{1\to 2}}} \\ 0 & 0 & 0.9519 \\ 0 & 1 & 0.9519\varepsilon \\ 0 & 2 & 0.9519\varepsilon \\ 1 & 0 & 0.0481\varepsilon \\ 1 & 1 & 0.0481 \\ 1 & 2 & 0.0481\varepsilon \\ 2 & 0 & 1.11\text{e-}8\varepsilon \\ 2 & 1 & 1.11\text{e-}8\varepsilon \\ 2 & 2 & 1.11\text{e-}8 \end{pmatrix}$$

$$= \begin{pmatrix} Y_{\mathbf{n_1}} & \tau_1 \\ 0 & 0.9519 + (0.0481 + 1.11\text{e-}8)\varepsilon \\ 1 & 0.0481 + (0.9519 + 1.11\text{e-}8)\varepsilon \\ 2 & 1.11\text{e-}8 + (0.9519 + 0.0481)\varepsilon \end{pmatrix} \overset{\varepsilon=1\text{e-}7}{\approx} \begin{pmatrix} Y_{\mathbf{n_1}} & \tau_1 \\ 0 & 0.9519 \\ 1 & 0.0481 \\ 2 & 1.11\text{e-}7 \end{pmatrix}.$$

Next, we eliminate variable $Y_{\mathbf{a_{3\to 1}}}$:

$$P(Y_{\mathbf{n_1}}) = \frac{1}{Z} \varphi_{\mathbf{n_1}}(Y_{\mathbf{n_1}}) \cdot \tau_1(Y_{\mathbf{n_1}}) \cdot \underbrace{\sum_{Y_{a_{3\to 1}}} \varphi_{\mathbf{a_{3\to 1}}}(Y_{\mathbf{a_{3\to 1}}}) \cdot \varphi_{\text{flow}}(Y_{\mathbf{a_{3\to 1}}}, Y_{\mathbf{n_1}})}_{\tau_2(Y_{\mathbf{n_1}})}$$

Similarly, we obtain for τ_2:

$$\begin{pmatrix} Y_{\mathbf{n_1}} & \tau_2 \\ 0 & 0.6902 + (0.3098 + 2.19\text{e-}7)\varepsilon \\ 1 & 0.3098 + (0.6902 + 2.19\text{e-}7)\varepsilon \\ 2 & 2.19\text{e-}7 + (0.6902 + 0.3098)\varepsilon \end{pmatrix} \overset{\varepsilon=1\text{e-}7}{\approx} \begin{pmatrix} Y_{\mathbf{n_1}} & \tau_2 \\ 0 & 0.6902 \\ 1 & 0.3098 \\ 2 & 3.19\text{e-}7 \end{pmatrix}.$$

Note that the beliefs in τ_i are very similar to the prior beliefs on the arcs because we chose ε very small. Finally, we multiply all remaining factors that depend only on the variable of interest and normalise the result:

$$P(Y_{\mathbf{n_1}}) = \frac{1}{Z} \begin{pmatrix} Y_{\mathbf{n_1}} & \varphi_{\mathbf{n_1}} \\ 0 & 0.9762 \\ 1 & 0.0238 \\ 2 & 5.37\text{e-}9 \end{pmatrix} \cdot \begin{pmatrix} Y_{\mathbf{n_1}} & \tau_1 \\ 0 & 0.9519 \\ 1 & 0.0481 \\ 2 & 1.11\text{e-}7 \end{pmatrix} \cdot \begin{pmatrix} Y_{\mathbf{n_1}} & \tau_2 \\ 0 & 0.6902 \\ 1 & 0.3098 \\ 2 & 3.19\text{e-}7 \end{pmatrix} = \frac{1}{Z} \begin{pmatrix} Y_{\mathbf{n_1}} & \varphi_{\mathbf{n_1}} \\ 0 & 0.6414 \\ 1 & 3.54\text{e-}4 \\ 2 & 1.90\text{e-}22 \end{pmatrix} = \begin{pmatrix} Y_{\mathbf{n_1}} & \varphi_{\mathbf{n_1}} \\ 0 & 0.9995 \\ 1 & 5.48\text{e-}4 \\ 2 & 2.93\text{e-}22 \end{pmatrix}.$$

Because all three singleton factors put a high belief in a multiplicity 0 for $\mathbf{n_1}$, we observe a strong belief in $Y_{\mathbf{n_1}} = 0$ in the final probability.

Finding the optimal order in which variables should be eliminated is NP-hard. We always eliminate the variable that has the fewest neighbours (and hence results in the smallest possible intermediate factor). Although this ordering is not guaranteed to be optimal, it works well in practice.

2 Parameter estimation formulas

The singleton factor parameters that we need to determine in the M-step of stage 2 are the following: the means of the negative binomial distributions λ and λ_0, the overdispersion factors f and f_0 that determine the variance of the negative binomials, and the weights w_i for all multiplicities. To calculate the values of these parameters we use the following values, derived from the data: we denote the (quality score weighted) k-mer coverage of a node or $(k+1)$-mer coverage of an arc as $\text{cov}(n)$ and $\text{cov}(a)$ respectively. Note that we are using a de Bruijn graph representation where linear chains of nodes are concatenated to unitigs; $\text{cov}(n)$ will thus be the average coverage of all k-mers in the concatenated node. The current multiplicity estimate for nodes and arcs is denoted by $\text{mult}(n)$ and $\text{mult}(a)$ respectively. Additionally, we will denote the soft multiplicity assignments obtained during the E-step as $\pi_m(n) = P(\text{mult}(n) = m)$ for nodes and $\pi_m(a) = P(\text{mult}(a) = m)$ for arcs. Since nodes correspond to (concatenated) k-mers, while the arcs correspond to $(k+1)$-mers, the underlying distributions will differ slightly. This is why all parameters are estimated separately for the nodes and the arcs of the de Bruijn graph.

To estimate the parameters of the negative binomial error distribution we fit a negative binomial distribution to count data generated by binning the observed q-mer coverages as follows:
For coverage $c \in \mathbb{N}$:

$$\text{count}_{\text{node}}(\text{bin}_c) = \sum_{\text{nodes } n} P(\text{mult}(n) = 0) \left[\mathbf{1}(\lfloor \text{cov}(n) \rfloor = c)(1 - \pi_n) + \mathbf{1}(\lfloor \text{cov}(n) \rfloor + 1 = c)\pi_n\right],$$

$$\text{count}_{\text{arc}}(\text{bin}_c) = \sum_{\text{arcs } a} P(\text{mult}(a) = 0) \left[\mathbf{1}(\lfloor \text{cov}(a) \rfloor = c)(1 - \pi_a) + \mathbf{1}(\lfloor \text{cov}(a) \rfloor + 1 = c)\pi_a\right],$$

where $\mathbf{1}(x)$ is an indicator function such that $\mathbf{1}(x) = 1$ if x is true and $\mathbf{1}(x) = 0$ otherwise and $\pi_n = \text{cov}(n) - \lfloor \text{cov}(n) \rfloor$, $\pi_a = \text{cov}(a) - \lfloor \text{cov}(a) \rfloor$. As we are dealing with truncated data due to unobserved zero-counts as well as the removal of low coverage nodes and arcs, we use an EM-procedure as follows:

- in the E-step we estimate pseudo-counts for the unobserved coverage values $c \in \{0, 1, \ldots, T\}$ (T the pre-processing coverage threshold) as

$$\text{count}_{\text{node}}(\text{bin}_c) = w_0 P_{Nb(\hat{\lambda}_0^{\text{node}}, \hat{f}_0^{\text{node}})}(\text{cov}(n) = c),$$
$$\text{count}_{\text{arc}}(\text{bin}_c) = w_0 P_{Nb(\hat{\lambda}_0^{\text{arc}}, \hat{f}_0^{\text{arc}})}(\text{cov}(a) = c)$$

- in the M-step we estimate the weight parameter (w_0) and the mean ($\hat{\lambda}_0$) and overdispersion factor ($\hat{f}_0$) of the negative binomial distribution based on the observed and pseudo counts as

$$w_0^{\text{node}} = \sum_{c=0}^{c_{\max}} \text{count}_{\text{node}}(\text{bin}_c) \qquad w_0^{\text{arc}} = \sum_{c=0}^{c_{\max}} \text{count}_{\text{arc}}(\text{bin}_c) \tag{1}$$

$$\hat{\lambda}_0^{\text{node}} = \frac{\sum_{c=0}^{c_{\max}} c \cdot \text{count}_{\text{node}}(\text{bin}_c)}{\sum_{c=0}^{c_{\max}} \text{count}_{\text{node}}(\text{bin}_c)}, \qquad \hat{\lambda}_0^{\text{arc}} = \frac{\sum_{c=0}^{c_{\max}} c \cdot \text{count}_{\text{arc}}(\text{bin}_c)}{\sum_{c=0}^{c_{\max}} \text{count}_{\text{arc}}(\text{bin}_c)}, \tag{2}$$

$$\hat{S}_0^{\text{node}} = \frac{\sum_{c=0}^{c_{\max}} \text{count}_{\text{node}}(\text{bin}_c)(c - \hat{\lambda}_0^{\text{node}})^2}{\sum_{c=0}^{c_{\max}} \text{count}_{\text{node}}(\text{bin}_c)}, \qquad \hat{S}_0^{\text{arc}} = \frac{\sum_{c=0}^{c_{\max}} \text{count}_{\text{arc}}(\text{bin}_c)(c - \hat{\lambda}_0^{\text{arc}})^2}{\sum_{c=0}^{c_{\max}} \text{count}_{\text{arc}}(\text{bin}_c)}, \tag{3}$$

$$\hat{f}_0^{\text{node}} = \frac{\hat{S}_0^{\text{node}}}{\hat{\lambda}_0^{\text{node}}}, \qquad \hat{f}_0^{\text{arc}} = \frac{\hat{S}_0^{\text{arc}}}{\hat{\lambda}_0^{\text{arc}}} \tag{4}$$

The E and M step are iterated until the parameter estimates do not change significantly anymore.

For the negative binomial distributions representing the true nodes and arcs we determine the method of moments estimators for mean and variance for each multiplicity (up until a certain multiplicity m' (tuneable)) after which we determine $\hat{\lambda}$ and $\hat{f}$ as a weighted average of these estimates. More specifically, the negative binomial parameters are estimated as follows:

$$w_m^{\text{node}} = \sum_{\text{nodes } n} \pi_m(n), \qquad w_m^{\text{arc}} = \sum_{\text{arcs } a} \pi_m(a), \tag{5}$$

$$\hat{\lambda}^{\text{node}} = \frac{\sum_{m=1}^{m'} \frac{1}{m} \sum_{\text{nodes } n} \pi_m(n)\text{cov}(n)}{\sum_{m=1}^{m'} w_m^{\text{node}}}, \qquad \hat{\lambda}^{\text{arc}} = \frac{\sum_{m=1}^{m'} \frac{1}{m} \sum_{\text{arcs } a} \pi_m(a)\text{cov}(n)}{\sum_{m=1}^{m'} w_m^{\text{arc}}}, \tag{6}$$

$$\hat{S}_m^{\text{node}} = \frac{1}{w_m^{\text{node}}} \sum_{\text{nodes } n} \pi_m(n) \left(\text{cov}(n) - m\hat{\lambda}^{\text{node}}\right)^2, \qquad \hat{S}_m^{\text{arc}} = \frac{1}{w_m^{\text{arc}}} \sum_{\text{arcs } a} \pi_m(a) \left(\text{cov}(a) - m\hat{\lambda}^{\text{arc}}\right)^2, \tag{7}$$

$$\hat{f}^{\text{node}} = \frac{1}{\sum_{m=0}^{m'} w_m^{\text{node}}} \sum_{m=0}^{m'} w_m^{\text{node}} \frac{\hat{S}_m^{\text{node}}}{m\hat{\lambda}^{\text{node}}}, \qquad \hat{f}^{\text{arc}} = \frac{1}{\sum_{m=0}^{m'} w_m^{\text{arc}}} \sum_{m=0}^{m'} w_m^{\text{arc}} \frac{\hat{S}_m^{\text{arc}}}{m\hat{\lambda}^{\text{arc}}}. \tag{8}$$

Note that the weights w_m are calculated up until m', while we might need weights for higher order multiplicities during the E-step. We define $w_m = w_{m'}, \forall m > m'$.

3 Confusion matrices

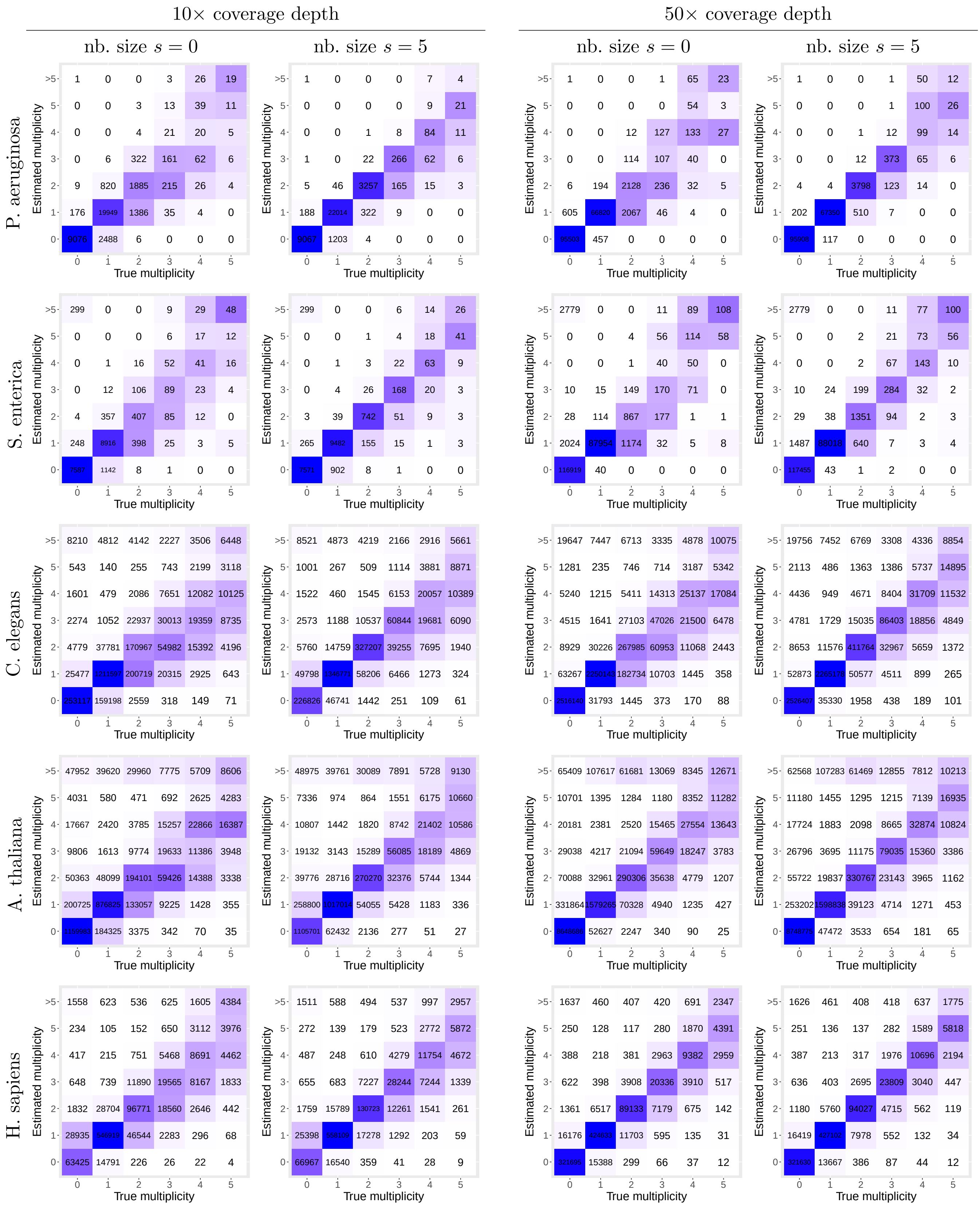

Table 1: Confusion matrices for different organisms (real data), coverage depths (10× and 50×) and neighbourhood sizes ($s = 0$ and $s = 5$). Each column contains all nodes with a specific true multiplicity and shows the distribution of the estimated multiplicities. For *H. sapiens*, 10^6 nodes were randomly sampled from the graph.

4 Number of EM-iteration per neighbourhood size

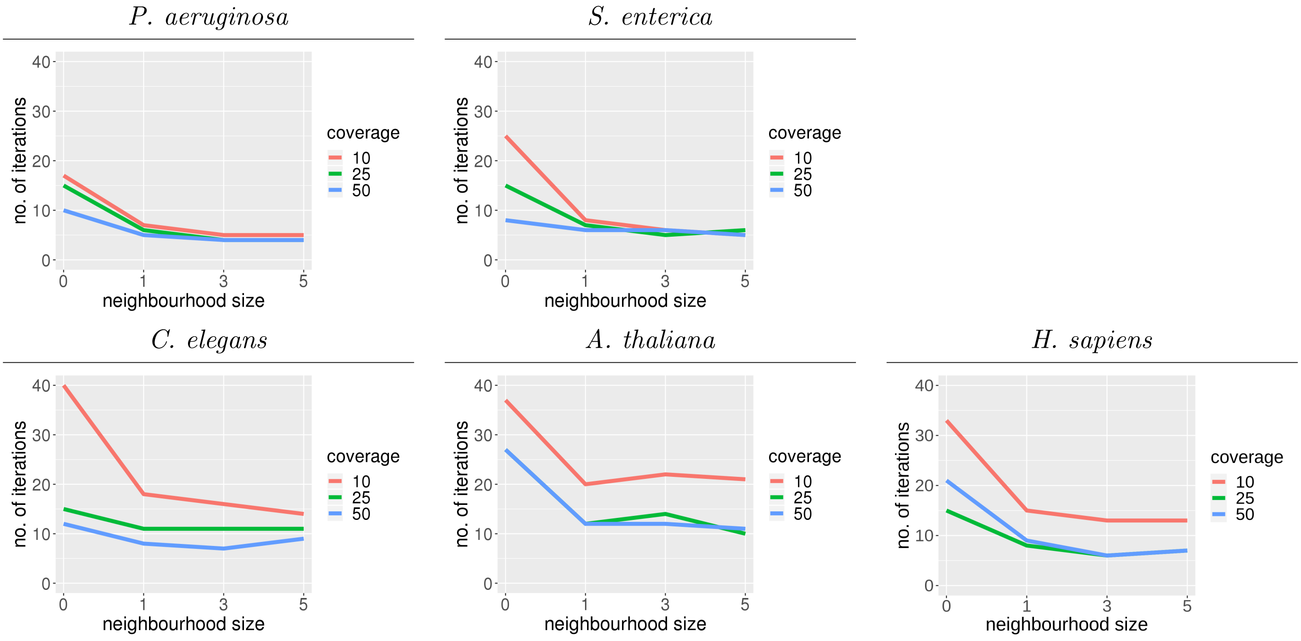

Table 2: Number of iterations in step 2 until the EM-procedure converged for 5 different organisms (real data), at different coverage depths and for different neighbourhood sizes ($s \in \{0, 1, 3, 5\}$). The maximum number of iterations was set to 40 for all tests.

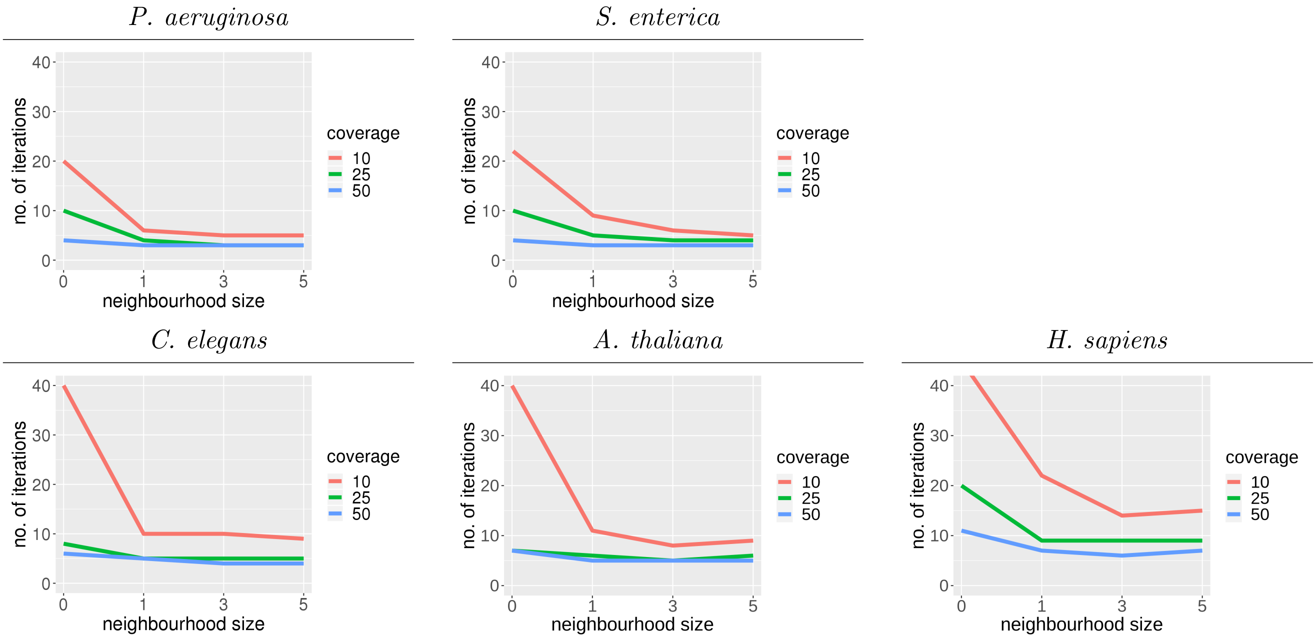

Table 3: Number of iterations in step 2 until the EM-procedure converged for 5 different organisms (simulated data), at different coverage depths and for different neighbourhood sizes ($s \in \{0, 1, 3, 5\}$). For the H. sapiens simulated data tests the maximum number of iterations was set tot 50. For all other simulated data tests the maximum number of iterations was set to 40.

5 Simulated data

Table 4: Estimation of the node multiplicity in de Bruijn graphs ($k = 21$) built from simulated Illumina data for 5 organisms (2 bacteria, 3 eukaryotes). Datasets of three different coverage depths were simulated: i.e. 10×, 25× and 50× coverage. For *H. sapiens*, the multiplicity was inferred for one million randomly sampled nodes; for all other datasets the multiplicity was inferred for all nodes. The node (resp. k-mer) accuracy refers to the percentage of nodes (resp. k-mers) in the de Bruijn graph that were assigned the correct multiplicity. The accuracy improves when using CRFs with increasing neighbourhood size s.

		10×		25×		50×	
	s	node acc.	k-mer acc.	node acc.	k-mer acc.	node acc.	k-mer acc.
P. aeruginosa	0	82.62	98.76	95.15	99.68	98.87	99.90
	1	92.90	99.49	98.90	99.93	99.73	99.98
	3	93.89	99.51	99.32	99.96	99.81	99.99
	5	94.31	99.53	99.59	99.99	99.87	99.99
S. enterica	0	76.79	99.37	93.48	99.81	97.76	99.89
	1	83.87	99.44	96.97	99.92	98.50	99.95
	3	85.45	99.34	97.60	99.93	98.75	99.96
	5	85.92	99.50	98.09	99.95	98.90	99.96
C. elegans	0	73.67	94.58	87.75	98.51	94.49	99.45
	1	84.66	97.51	92.52	99.22	96.29	99.70
	3	87.01	98.13	93.54	99.39	96.71	99.76
	5	87.49	98.25	93.84	99.44	96.89	99.78
A. thaliana	0	71.29	94.39	86.01	98.45	93.41	99.4
	1	82.36	97.65	90.90	99.23	95.27	99.68
	3	83.79	98.02	92.00	99.40	95.79	99.74
	5	84.44	98.09	92.55	99.48	96.06	99.77
H. sapiens	0	75.45	92.03	87.43	96.79	94.23	98.95
	1	83.61	95.97	91.75	98.38	95.88	99.36
	3	84.91	96.52	92.53	98.64	96.26	99.44
	5	85.27	96.68	92.78	98.74	96.38	99.47

6 Breakdown of error types

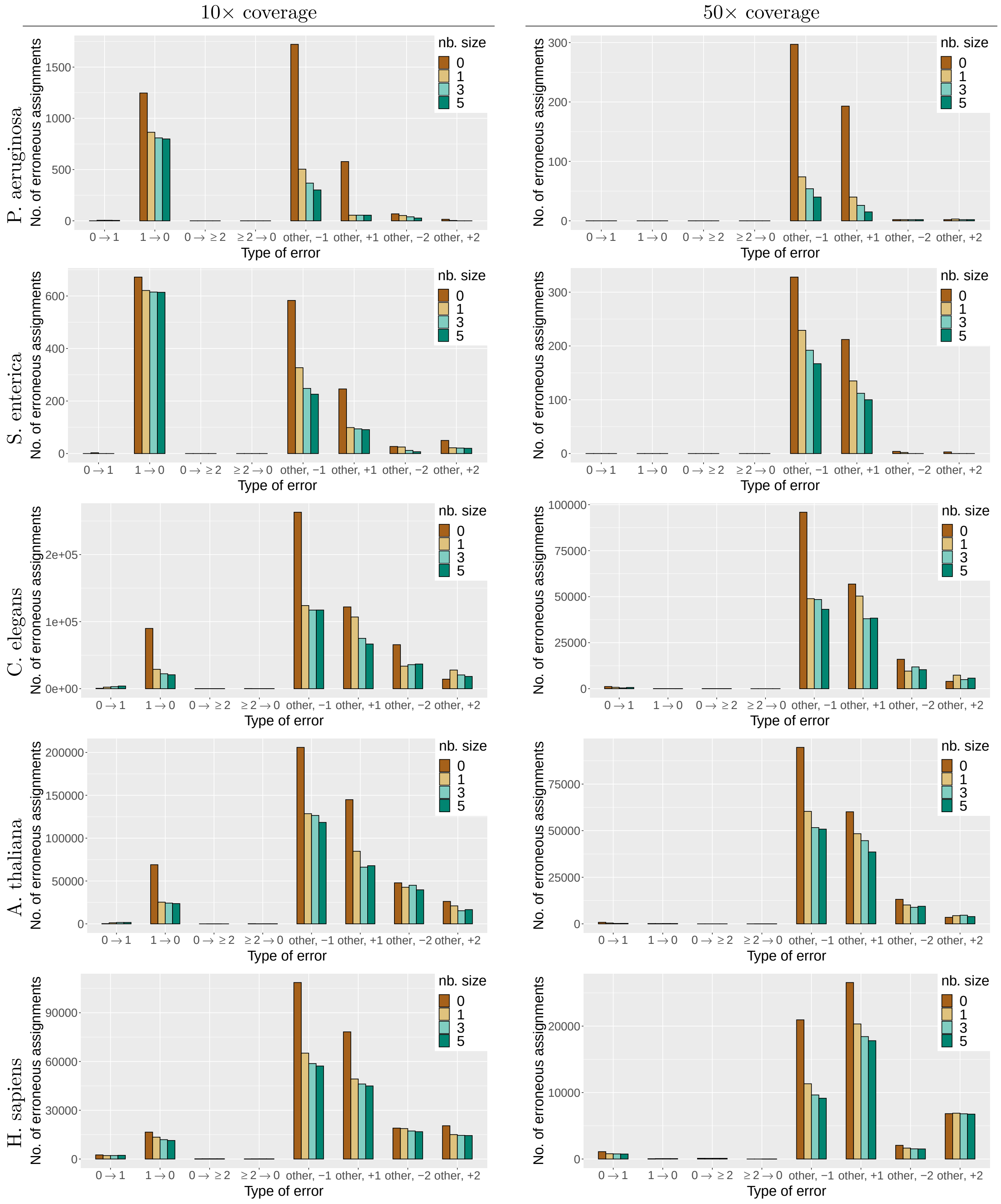

Table 5: Breakdown of error types for different organisms (simulated data), coverage depths (10× and 50×) and neighbourhood sizes ($s \in \{0, 1, 3, 5\}$). The different classes of errors are: a) sequencing error labelled as unique region ($0 \to 1$); b) unique region labelled as sequencing error ($1 \to 0$); c) repeated region labelled as sequencing error ($\geq 2 \to 0$); d) sequencing error labelled as repeated region ($0 \to \geq 2$); e) other nodes labelled one too low (other, -1); f) other nodes labelled one too high (other, $+1$); g) other nodes label more than one too low (other, -2); h) other nodes labelled more than one too high (other, $+2$).

7 Influence of parameter values

Based on simulated datasets we explore the influence of several parameters that can be user-defined in our pipeline. All experiments were run for two different neighbourhood sizes, 3 and 6, as well as for neighbourhood size 0, which determines multiplicities based on the negative binomial mixture model alone.

The 'flow conservation strength' parameter is defined as $f = \frac{1}{\varepsilon}$, with ε the value that the flow factors assign to a multiplicity combination for which conservation of flow does not hold. Figure 1 shows the accuracy obtained by using different values for this parameter, i.e. $f = 10, 10^3, 10^5, 10^7, 10^9$. To obtain these results, we used the full set of nodes and arcs to train the model in stage 2, such that there was no variance possible due to different subsets for training. As can be seen in Figure 1, multiplicity determination accuracy increases as we impose a higher 'flow conservation strength'. However, from values $= 10^5$ and higher the accuracy gain is minimal. Based on these results we set the default value for this parameter to 10^7.

The influence of the size s of the subset used for EM training on variability in negative binomial parameter estimation, and on the accuracy obtained in stage 3 is shown in Figures 2 and 3. We performed EM-training with subsets of $100, 1000, 10^4$ and 10^5 nodes and arcs. From these results we conclude that it is not necessary to train EM on the full dataset to get consistent multiplicity assignments in stage 3. We can choose a subset size small enough such that the time-consumption for the whole pipeline is dominated by stage 3, while still obtaining negative binomial parameter estimates that are good representatives for the whole dataset.

References

[1] Koller, D., Friedman, N.: Probabilistic Graphical Models: Principles and Techniques. MIT press, Cambridge, Massachusetts (2009)

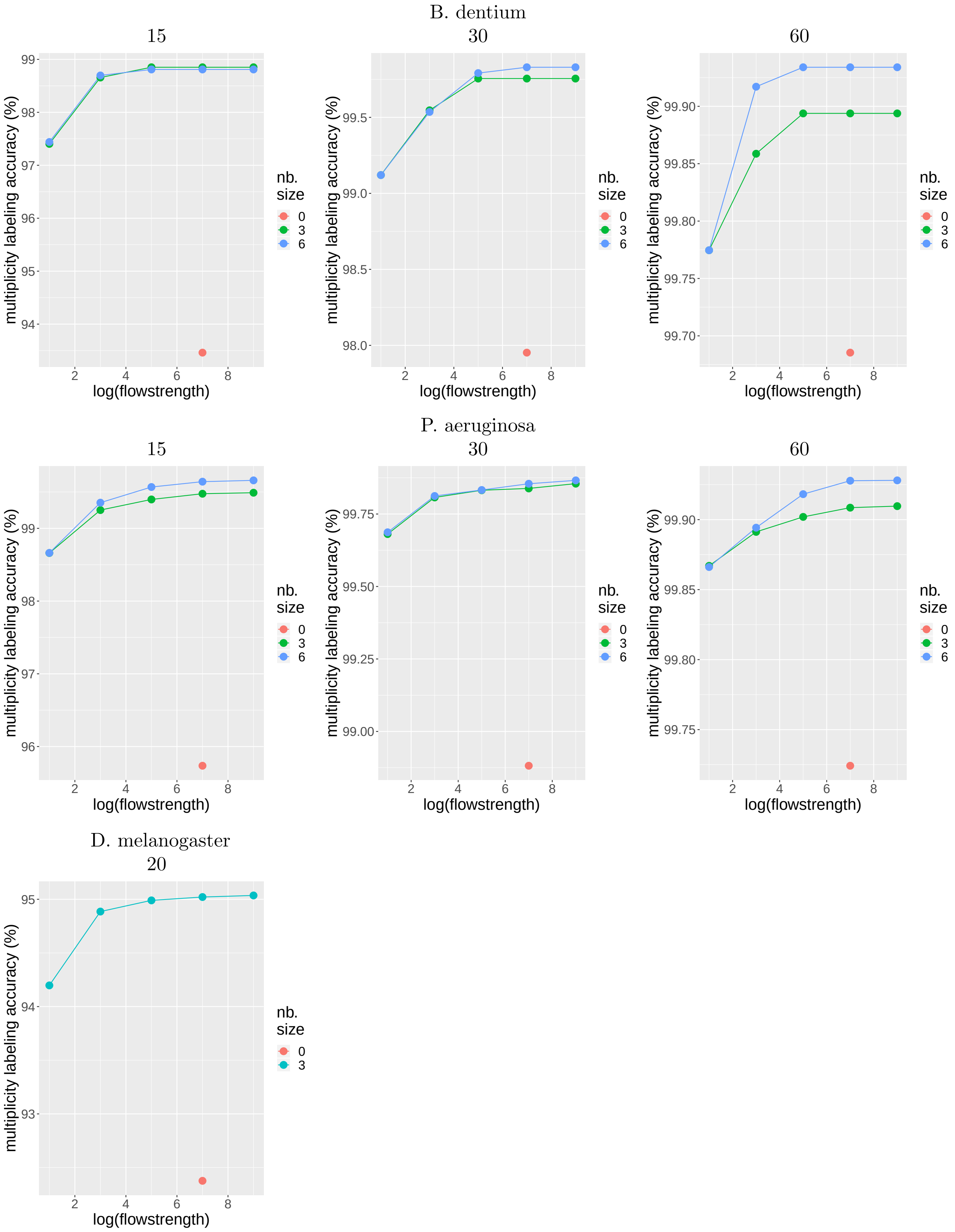

Figure 1: Influence of conservation of flow strength parameter on final accuracy reached. Results are given for 2 simulated bacterial datasets (B. dentium and P. aeruginosa) for 3 different coverages and for 1 simulated eukaryote dataset (D. melanogaster) at 20× coverage.

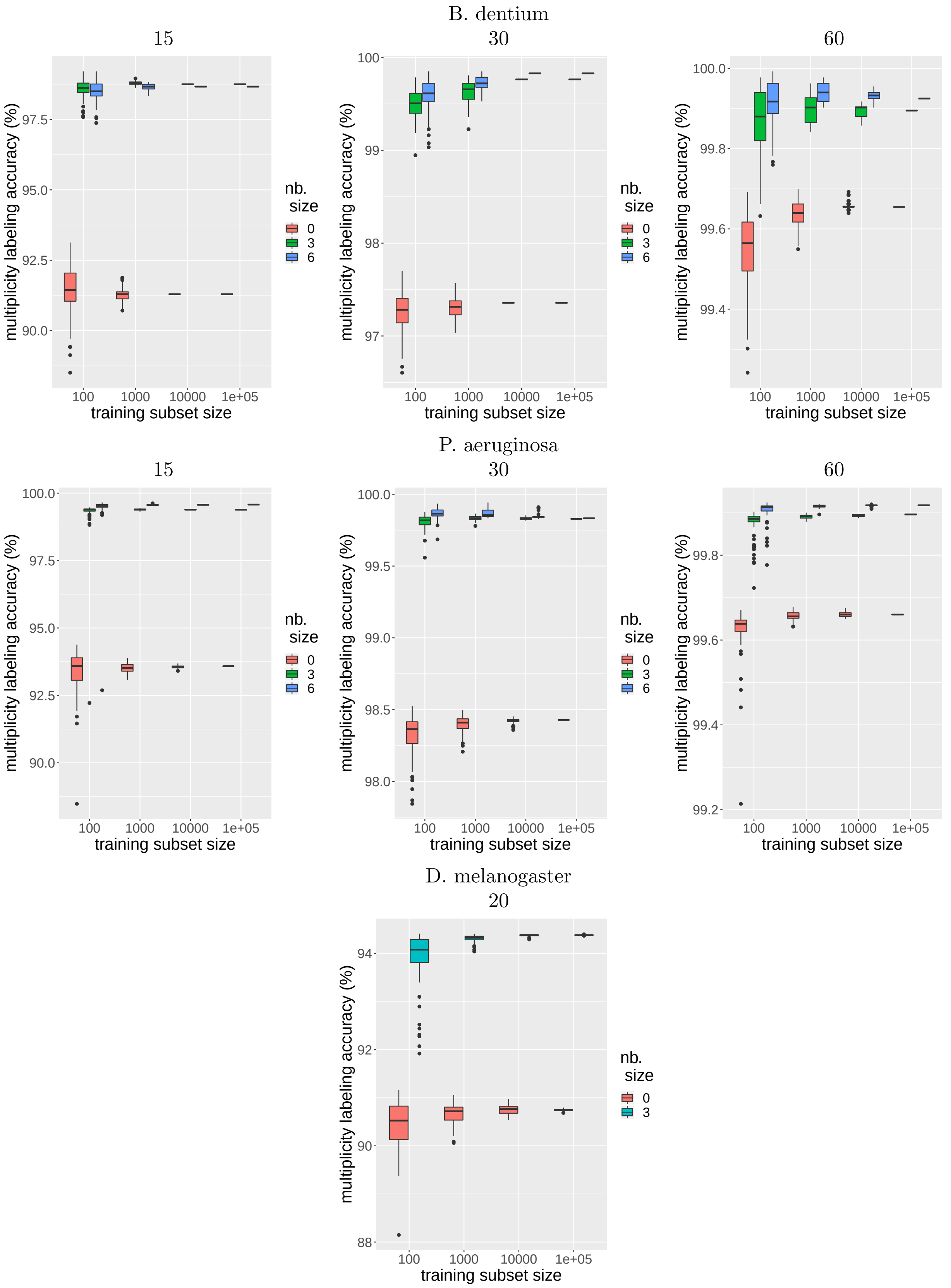

Figure 2: Influence on the final accuracy of the size of the subset used for EM-training of the parameters in stage 2 of our pipeline. Results are given for 2 simulated bacterial datasets (B. dentium and P. aeruginosa) for 3 different coverages and for one eukaryote dataset (D. melanogaster) at 20× coverage.

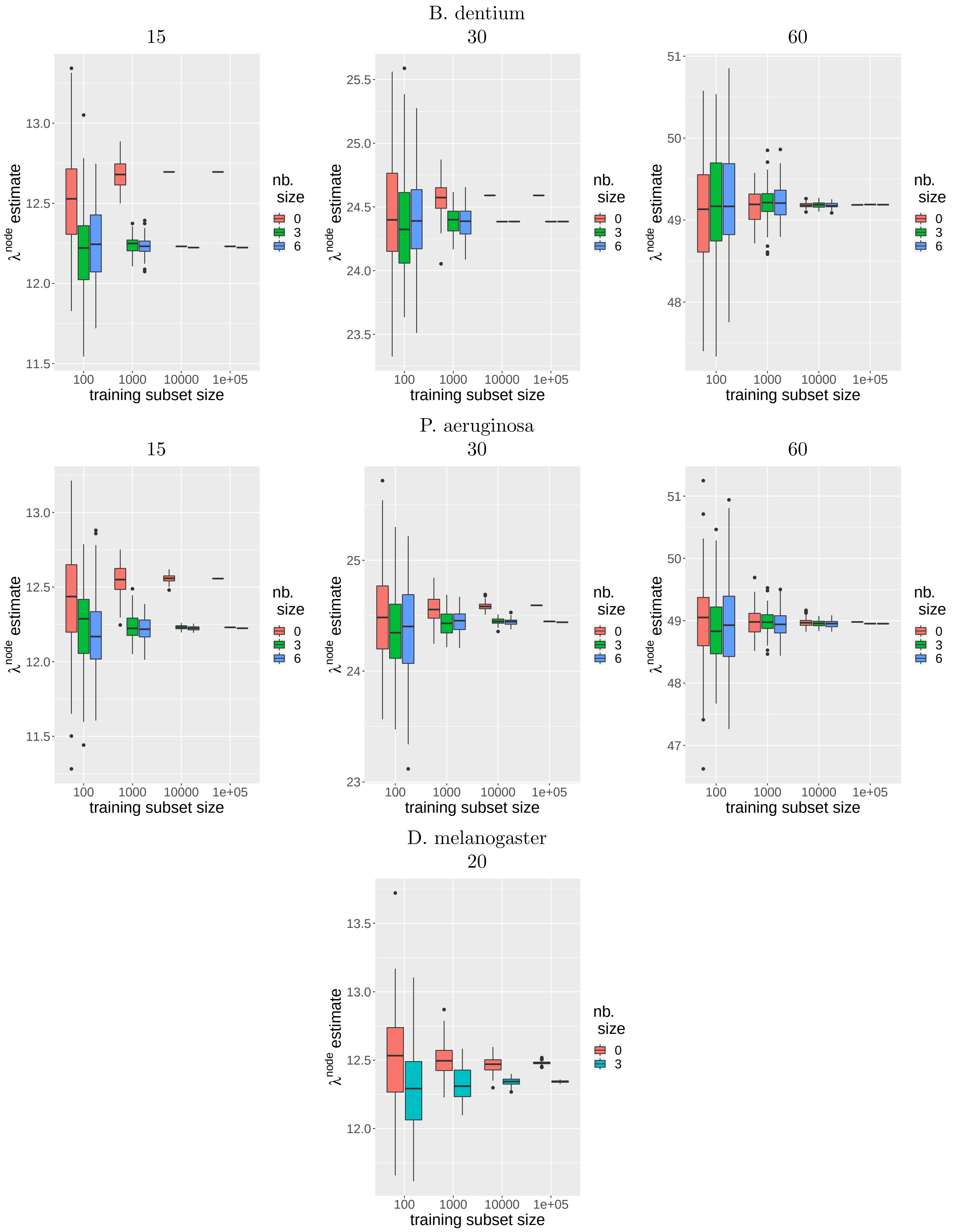

Figure 3: Influence on negative binomial parameter estimates of the size of the subset used for EM-training of the parameters in stage 2 of our pipeline. Results are given for 2 simulated bacterial datasets (B. dentium and P. aeruginosa) for 3 different coverages and for one eukaryote dataset (D. melanogaster) at 20× coverage.
